# Home-based exercise and support programme for people with dementia and their caregivers: study protocol of a randomised controlled trial

**DOI:** 10.1186/1471-2458-11-894

**Published:** 2011-11-25

**Authors:** Anna-Eva Prick, Jacomine de Lange, Erik Scherder, Anne Margriet Pot

**Affiliations:** 1 Department of Clinical Psychology, VU University Amsterdam, Van der Boechorststraat 1, 1081 BT Amsterdam, The Netherlands; 2 Programme on Ageing, Netherlands Institute on Mental Health and Addiction, Da Costakade 45, 3500 AS Utrecht, The Netherlands; 3 Department of Clinical Neuropsychology, VU University Amsterdam, Van der Boechorststraat 1, 1081 BT Amsterdam, The Netherlands; 4 Mental Health, EMGO+-Institute: Institute for Health and Care Research, Van der Boechorststraat 7, 1081 BT Amsterdam, The Netherlands; 5 Rotterdam University, Museumpark 40, 3015 CX Rotterdam, The Netherlands

## Abstract

**Background:**

Dementia affects the mood of people with dementia but also of their caregivers. In the coming years, the number of people with dementia will increase worldwide and most of them will continue to live in the community as long as possible. Home-based psychosocial interventions reducing the depressive symptoms of both people with dementia and their caregivers in their own home are highly needed.

**Methods/Design:**

This manuscript describes the design of a Randomised Controlled Trial (RCT) of the effects of a home-based exercise and support programme for people with dementia and their caregivers. The aim is to randomly assign 156 dyads (caregiver and dementia diagnosed person) to an intervention group or a comparison group. The experimental group receives a home programme in which exercise and support for the people with dementia and their caregivers are combined and integrated. The comparison group receives a minimal intervention. Primary outcomes are physical health (people with dementia) and mood (people with dementia and caregivers). In addition, to get more insight in the working components of the intervention and the impact of the intervention on the relationship of the dyads a qualitative sub-study is carried out.

**Discussion:**

This study aims to contribute to an evidence-based treatment to reduce depressive symptoms among people with dementia and their caregivers independently living in the community.

**Trial Registration:**

The study has been registered at the Netherlands National Trial Register (NTR), which is connected to the International Clinical Trials Registry Platform of the WHO. Trial number: NTR1802.

## Background

Dementia affects the mood of people with dementia but also of their family caregivers [1,2]. Psychosocial interventions have been demonstrated to reduce caregiver distress and delay nursing home admission [3]. However, benefits of psychological interventions on caregiver's psychological mental health are limited especially with regard to caregivers' depressive symptoms [4-6]. Since the number of people with dementia is increasing worldwide, and most of them will continue to live in the community as long as possible, home-based psychosocial interventions reducing the depressive symptoms of both people with dementia and their caregivers in their own home are highly needed.

A review on combined programmes for people with dementia and their caregivers showed the effectiveness of four out of the 22 programmes for couples [7]. Two of these programmes showed significant beneficial effects on the depressive symptoms of persons with dementia as well as their caregivers: a short-term residential programme [8] and an active behavioral treatment developed by Teri [9]. Another intervention of Teri showed a reduction of depressive symptoms of people with dementia and an improvement of their physical functioning [10]. This intervention combines a physical exercise programme with teaching caregivers behavioral management techniques and to identify pleasant activities. The impact on caregivers' mental health was not measured in this study.

There is more research supporting the beneficial effects of physical exercise on the cognitive, physical and behavioural functioning of people with dementia [11-14]. Since physical exercise in combination with behavioral activation and pleasant activities seems to be promising and can be easily provided in the home of the people with dementia, an intervention programme like the one studied by Teri might have opportunities in other countries outside the US as well. Furthermore, it might have a beneficial effect on the relationship between persons with dementia and their caregivers. Since pre-caregiving and current relationship quality between care receiver and caregiver appear to have an impact on caregiver's wellbeing [15], this might be an additional advantage of such a combined intervention programme.

### Aim and main research questions

This study aims to develop an evidence-based treatment to reduce depressive symptoms among home living people with dementia and their caregivers. The feasibility and effectiveness of physical exercise and behavioural management and activation on depressive symptoms of both people with dementia and their caregivers will be investigated. A Randomised Controlled Trial (RCT) and a qualitative sub study are conducted to examine the effects of this programme. This article describes the protocol and discusses its benefits en limitations.

The main research questions are:

1. Is the home-based exercise and support programme feasible?

2. What is the effect of a home-based exercise and support programme on physical health, mood, cognitive functioning and behaviour of people with dementia?

3. What is the effect of the home-based exercise and support programme on general health, feelings of burden and mood of caregivers?

4. What are the working components of the intervention as perceived by the people with dementia and the caregivers?

5. What is the impact of this intervention on the quality of the relationship between the person with dementia and his/her caregiver?

## Methods/Design

### Participants

All dyads willing to participate in the study are eligible. Inclusion criteria for people with dementia are a diagnosis of dementia made by a physician (a general practitioner or a neurologist), minimum age 55 years, living at home with a caregiver willing to participate in the training sessions. Exclusion criteria concerning the people with dementia are the use of antidepressants, the presence of psychotic symptoms, Mini Mental State Examination (MMSE) score < 14 and receiving more than 2 days respite care in a day care facility.

Caregivers are spouses or adult relatives who live with the care receiver or spend a minimum of 4 hours every day with the person with dementia. Furthermore, caregivers should have a Centre for Epidemiologic Studies-Depression (CES-D) score > 5 and should have enough understanding of the Dutch language. Exclusion criteria for the caregivers are physical disorders that hamper assistance with the exercises, presence of psychotic symptoms and use of antidepressants.

### Sample size/Power

Sample sizes were calculated by a statistical power analysis. To detect an effect size of *d *> 0.40 between the two conditions with α = .05 and β = .80, 78 dyads in each group are needed.

### Recruitment

Participants for this study are recruited throughout the Netherlands via caregiver centers, Alzheimer Cafés (easily accessible meetings for people with dementia, their caregivers and others) and by advertising in newspapers and via the Internet.

### Procedure

Trained master students in clinical psychology screen the interested dyads at intake. Eligible dyads are invited for baseline assessment and asked to sign an informed consent. When necessary, caregivers provide consent on behalf of the person with dementia. After the baseline assessment dyads are randomly allocated to the intervention or the comparison group. Within 2 weeks after the baseline measurement the intervention starts.

### Randomisation

After the baseline assessment dyads are randomly assigned by blocked randomisation (block size 20) to the intervention or comparison group. The allocation schedule is made by an independent researcher with a computer generated block randomisation. In this RCT dyads are aware of the treatment assigned. The examiners are blinded to the group allocation.

### Intervention group: combined home-based exercise and support programme

The intervention group receives a programme consisting of two parts: an exercise programme and a support programme. The goal of the exercise programme is to motivate dyads to complete 30 min of active exercise at least 3 days a week. There are four exercise components in the programme: flexibility, strengthening, balance and endurance. The activities should be fun and the person with dementia should enjoy performing them. The exercises are introduced gradually, session-by-session. The caregiver assists the person with dementia with the exercises at his/her level of physical abilities. Caregivers should watch carefully to prevent falls. If the person with dementia develops any sudden pain, shortness of breath, or ill feelings, exercises should be discontinued and a physician should be consulted. In addition, the goal of the support programme is to train caregivers in skills to communicate with the person with dementia and to stimulate implementation of pleasant activities in daily live. A coach visits the dyads in their own homes for 8 one-hour-long sessions during 3 months. The first month the dyads are visited weekly, followed by biweekly sessions over the next 8 weeks. The coaches are trained master students of the VU University at the Department of Clinical Psychology who follow a special training programme on geropsychology. During the study, all trained students receive minimal 3 times supervision of a psychologist specialized in working with older people.

For the present study, the intervention of Linda Teri has been translated into Dutch. The transfer from the American intervention to the Dutch design involved several adaptations to the Dutch care situation. The present intervention also includes elements of a Dutch exercise programme for people with dementia and their caregivers [16]. Materials like a ball, weights and elastics were added to make the exercises more attractive for people with dementia. Finally, in view of the implementation of the intervention in daily practice the intervention program needed to be shortened. The number of sessions has been decreased to eight (instead of the original 12) and, in addition, the frequency of the sessions in the first month to one instead of 2 sessions per week. According to the caregivers in the pilot study, the frequency and number of sessions were too high, consuming too much of their valuable time.

### Comparison group: minimal intervention

The comparison group receives a minimal intervention consisting of 1) special information bulletins with general information (like car driving, medication, night rest and domotica) monthly sent to the dyads; and 2) monthly phone calls by the coaches. The goal of these 10 min phone calls is to encourage staying active in everyday life and to keep social contacts and to discuss the daily routine. After 6 months the dyads in the comparison group are offered the opportunity to participate in the home-based exercise and support programme.

### Measurements

**Figure 1:**
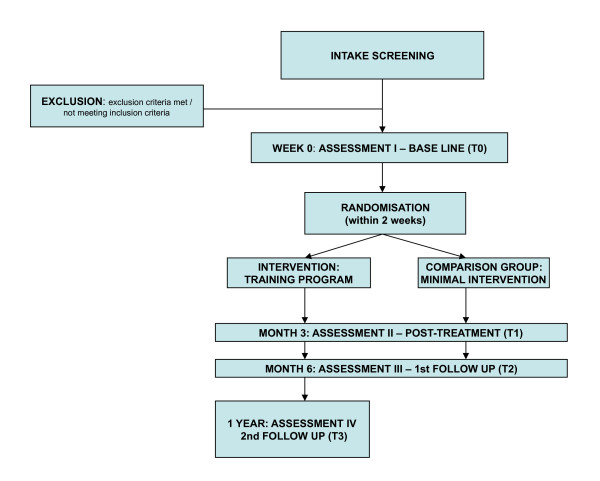
**Research procedure**.

Measurements take place at baseline, at the end of the intervention after 3 months (post treatment) and at 6 months after baseline (follow up). For the intervention group only, there is a second follow up 12 months after baseline to examine whether effects are still present (Figure 1; Tables 1 and 2).

**Table 1:** Instruments at different assessment moments for people with dementia

Outcomes	Measurements	T0	T1	T2	T3
**PRIMARY OUTCOMES**					
Physical health	SF-36	x	x	x	x
	SIP	x	x	x	x

Depressive symptoms	MDS-DRS RAI HC	x	x	x	x
	Cornell	x	x	x	x
	GDS-15	x	x	x	x

**SECONDARY OUTCOMES**					
Behavioural problems	RMBPC problems	x	x	x	x

Cognition	GDS	x			
	MMSE	x	x	x	x
	ADS-AWT	x	x	x	x
	BADS-Key Search	x	x	x	x
	TMT A & B	x	x	x	x
	RBMT FR & PR	x	x	x	x
	GIT-Category Fluency	x	x	x	x
	Digit Span FW & BW	x	x	x	x

Rest/Activity	Actigraphy	x	x		

APOE4	ApoE4	x			

*SF*-36, Short Form Health Survey, *SIP *Sickness Impact Profile, *MDS-DRS RAI HC *Depression Rating Scale of the Resident assessment Instrument Home Care, *Cornell *Cornell Scale for Depression in Dementia, *GDS*-15 Geriatric Depression Scale 15, *RMBPC *Revised Memory and Bahaviour Problem Checklist, *GDS *Global Deterioration Scale, *MMSE *Mini Mental State Examination, *ADS-AWT *8 Words Test of the Amsterdam Dementia Screeningtest, *BADS-Key Search *Key Search Test of the Behavioural Assessment of the Dysexecutive Syndrome, *TMT A & B *Trailmaking Test A & B, *RBMT *Face Recognition & Picture Recognition of the Rivermead Behavioral Memory Test, *GIT-Category Fluency *Category Fluency from the Groninger Intelligence Test, *Digit Span FW & BW *Digit Span Test (forward and backward) of the Wechsler Memory Scale, *ApoE4 *Apolipoprotein E4

**Table 2:** Instruments at different assessment moments for caregivers

Outcomes	Measurements	T0	T1	T2	T3
**PRIMARY OUTCOMES**					
Depressive symptoms	CES-D x	x	x	x	x

**SECONDARY OUTCOMES**					
General Health	GHQ	x	x	x	x

Burden	SPPIC	x	x	x	x
	RMBPC	x	x	x	x

Adrenocortical activity	Cortisol	x	x		

*CES-D *Centre for Epidemiologic Studies-Depression, *GHQ *General Health Question, *SPICC *Self-Perceived Pressure from Informal Care, *RMBPC *Revised Memory and Bahaviour Problem Checklist

### Instruments people with dementia

#### Primary outcome measures

##### Physical health and function

Two of the eight subscales of the Medical Outcome Study Short Form Health Survey (SF-36; [17,18]) are used to measure physical function and physical role functioning. Three subscales of the Sickness Impact Profile (SIP;[19,20]) are used to measure body care and movement, mobility and home management. Higher SF-36 scores indicate better health functioning; higher SIP scores indicate worse function. The caregiver completes both instruments.

##### Mood

The examiner completes the 19-item Cornell Scale for Depression in Dementia [21,22] together with the caregiver. The examiners are extensively trained and supervised by a psychologist to ensure interrater reliability. The Depression Rating Scale (DRS) of the Resident Assessment Instrument Home Care (RAI HC;[23-25]) is an observational scale consisting of seven items. Geriatric Depression Scale 15 (GDS 15) is used as a self-report measure [26,27]. Higher Cornell, DRS and GDS scores indicate greater impairment.

#### Secondary outcome measures

##### Behavioural problems

The Revised Memory and Behaviour Problem Checklist (RMBPC; [28,29]) is used to assess the level of behavioural disturbance of the person with dementia. The scale consists of 24 items reflecting three subscales, which measure the frequency of depressive behaviour, disruptive behaviour and memory related problems. Caregivers indicate how often problems occurred within the past week. Higher scores indicate greater disease severity.

##### Actigraphy rest/activity cycles

Rest activity data are collected by means of a wrist-worn, watch-size, ambulatory motion-detecting device, the Actigraph (Camebridge Neurotechnology Ltd., Camebridge, Great Britain). The Actigraph is set to record arm motion in 1-min epochs. People with dementia are asked to wear the Actigraph 24 h a day on their non-dominant wrist during 1 week. Data collected by Actigraph are related with rest-activity levels, an indirect measurement for sleep-wake cycles [30]. Interdaily Stability is a measure of the degree of resemblance between activity patterns of individual days. Higher values indicate a more stable rhythm. Intradaily Variability represents the fragmentation of periods of rest and activity. Normal rest-activity patterns show one major active period (day) and one major inactive period (night) and therefore show a low Intradaily Variability. Higher values indicate a more fragmented rhythm. Relative Amplitude represents the normalized difference between the most active 10-h period in the 24-h cycle in relation to the uninterrupted least active 5-h period. Higher values indicate a larger difference between daytime activity and night time rest and thus a stronger rhythm. The three rest-activity rhythm variables are converted into *z*-scores and combined to one Total Rest Activity Score.

##### Cognitive functioning

The *Global Deterioration Scale *(GDS; [31]) is used to clinically distinguish between the global stages from normality to severe dementia.

The *Mini Mental State Examination *(MMSE; [32,33]) is used to get an impression of global cognitive functioning.

The *8 Words Test *of the Amsterdam Dementia Screening Test 6 (ADS 6; [34]) is used to measure episodic anterograde memory.

To measure the ability to plan a strategy to solve a problem the *Key Search Test *of the Behavioural Assessment of the Dysexecutive Syndrome (BADS; [35]) is used.

*Trailmaking Test A & B *(TMT A & B; [36]) are used to measure visual conceptual and visuomotor tracking, flexibility and working memory.

The *Digit Span Test *(forward and backward) is part of the Wechsler Memory Scale-Revised (WMS-R; [37]). Work-memory is measured by digit span backward and short-term memory and attention is measured by digit span forward.

To measure visual long-term memory the *Face and Picture Recognition of the Rivermead Behavioural Memory Test *(RBMT; [38,39]) is used.

*Category Fluency *is a subtest from Amsterdam Dementia Screening Test 6 (ADS 6; [34]). This test requires a strategic search mechanism to retrieve information from semantic memory and is used to measure categorization abilities.

##### Apolipoprotein E4

The status of Apolipoprotein Epsilon 4 (ApoE4) is determined as a baseline variable and is taken from two buccal mucosa swabs (Catch-All swabs of the firm BIOzymTC example). ApoE4 might modify the effects of pharmacological treatments. An ApoE4 carrier may react different on a certain treatment, e.g. cholinesterase inhibitors [40]. To control for a possible influence of ApoE4 on treatment effects, we analyze ApoE4.

### Instruments caregivers

#### Primary outcome measure

##### Depressive symptoms

To measure depressive symptoms the self-rating 20-item report scale Centre for Epidemiologic Studies-Depression (CES-D; [41,42]) is used.

Higher scores indicate more severe depressive symptoms.

#### Secondary outcome measures

##### General health

One self-report question on general health is used. A higher score indicate higher impairment.

##### Burden

The Self-Perceived Pressure from Informal Care (SPPIC); [43,44] is applied to measure caregivers' feelings of role overload. Higher scores indicate more perceived pressure/feelings of role overload.

To assess caregiver distress the Revised Memory and Behaviour Problem Checklist (RMBPC) [28,29] is used. This is a 24-item caregiver-report measure of observable behavioural problems in people with dementia in relation to caregiver distress. Higher scores indicate higher caregiver distress.

##### Adrenocortical activity

Salivary cortisol is analyzed to measure adrenocortical activity. Several studies showed that salivary cortisol is a reliable reflection of cortisol levels in blood [45]. Cortisol is assessed by sampling saliva at the time of awakening and within the first 30 min after awakening. Early morning cortisol levels seems to be a biological marker for the individual's adrenocortical activity when measured with strict reference to the time of awakening [46].

#### Measurement of possible confounders

To collect dyads' demographic information and information about their health status questions are asked concerning nationality, birth date, living situation, education, ethic origin, use of medication, history of mental and physical health, present health status, Body Mass Index, use of alcohol and drugs, exercise history, dementia type, patient-caregiver relationship and care service use.

#### Statistical analysis

Data will be analyzed according to the intention-to-treat principle. Outcome analyses will compare the intervention group with the comparison group using regression analysis [47]. Baseline differences in demographic and clinical characteristics will be investigated using Chi-square tests, *t*-tests, and analysis of variance (ANOVA).

### Qualitative sub study

The qualitative part of this study provides more insight into the working components of the home-based intervention as perceived by the people with dementia and their caregivers. In addition, this qualitative study provides more insight into the impact of the intervention on the relationship of the dyads

## Methods

Semi-structured interviews are held with caregivers and people with dementia randomised to the intervention group. Consenting caregivers and people with dementia are individually interviewed at home. Caregivers and people with dementia with diverse sociodemographic characteristics are recruited. Recruitment continues until theoretical saturation is reached.

### Analysis

Interviews are audio taped and transcribed. The transcripts are initially coded using open codes based on the words the participants used. Coding starts after the first interview so that any emergent themes can be incorporated into subsequent interviews. The coding process is supported by ATLAS.ti (version 6.2; ATLAS.ti GmbH, Berlin, Germany). To ensure external validation and increase reliability, two researchers independently code the data. Differences are discussed until consensus is reached.

### Ethical principles

The study is conducted according to the principles of the Declaration of Helsinki and in accordance with the Medical Research Involving Human Subjects Act. The Medical Ethics Committee of the VU University Medical Center approved the study protocol (registration number 2008/320).

## Discussion

This paper describes the study protocol of a randomised controlled trial of a home-based physical exercise and support programme for people with dementia and their caregivers. Home-based psychosocial interventions for people with dementia and their caregivers are highly relevant, because in the coming years the number of people with dementia will increase and most of them will continue to live at home. Therefore, this study is aimed at the development of an evidence-based treatment of people with dementia and their caregivers in the community. Furthermore, a qualitative sub study is carried out to provide insight into the working components of the intervention as perceived by the people with dementia and the caregivers and to provide insight into the impact of this intervention on the quality of the relationship of the dyads.

A major strength of this study is the focus on both people with dementia and their caregivers. Both caregivers and people with dementia are included in the intervention and the potential outcomes of the intervention on both parties are assessed. We expect that both parties benefit from the intervention, both directly but also indirectly due to the positive effects on their counterpart. Another strength is the measurement of depressive symptoms from different perspectives with subjective and more objective measures to create a complete overview of mood in the dyads. Depressive symptoms of people with dementia are measured by clients' self-report (GDS-15) and caregivers' and clinical examiners' observation (Cornell and MDS-DRS RAI HC respectively). In addition, depressive symptoms of caregivers are measured by a self-report measure (CES-D) as well as by a biological marker (adrenocortical activity).

Designing a psychosocial intervention study also faces a number of challenges. First, it is impossible to blind coaches and the dyads for treatment conditions. The students that collect the data will be blinded to try to minimize the information bias effect. However, a risk is that the dyads disclose their group allocation during the assessments. Therefore, before each assessment dyads are asked not to inform the examiners about the intervention. To monitor the success or failure of the blinding, after each measurement the examiners are asked whether they know to which group the dyads had been allocated. Furthermore, the most appropriate control condition for this type of intervention could be debated. In this study, we chose to provide a minimal, but meaningful intervention to participants in the comparison group, rather than attention only. The minimal intervention consisted of written educational material and brief telephone calls.

This community-based intervention has the potential to improve the mental health of both people with dementia and their caregivers, which is highly relevant regarding the rising burden of dementia on society.

## Competing interests

The authors declare that they have no competing interests.

## Authors' contributions

A-EP coordinates the study, helped designing the study and wrote the manuscript. AMP is principle investigator and wrote the design of the study. JdL is member of the project-group and wrote the design of the study. ES is member of the project-group. All authors provided comments, read and approved the final manuscript.

## Pre-publication history

The pre-publication history for this paper can be accessed here:

http://www.biomedcentral.com/1471-2458/11/894/prepub

## References

[B1] Cuijpers P Depressive disorders in caregivers of dementia patients: a systematic review Aging Ment Health 2005 9 4 325 330 10.1080/13607860500090078 16019288

[B2] Cooper C Balamurali TB Selwood A Livingston G A systematic review of intervention studies about anxiety in caregivers of people with dementia Int J Geriatr Psychiatry 2007 22 3 181 188 10.1002/gps.1656 17006872

[B3] Olazaran J Reisberg B Clare L Cruz I Pena-Casanova J Del Ser T Woods B Beck C Auer S Lai C Nonpharmacological therapies in Alzheimer's disease: a systematic review of efficacy Dement Geriatr Cogn Disord 2010 30 2 161 178 10.1159/000316119 20838046

[B4] Selwood A Johnston K Katona C Lyketsos C Livingston G Systematic review of the effect of psychological interventions on family caregivers of people with dementia J Affect Disord 2007 101 1-3 75 89 10.1016/j.jad.2006.10.025 17173977

[B5] Thompson CA Spilsbury K Hall J Birks Y Barnes C Adamson J Systematic review of information and support interventions for caregivers of people with dementia BMC Geriatr 2007 7 18 10.1186/1471-2318-7-18 17662119 PMC1951962

[B6] Gatz M Commentary on evidence-based psychological treatments for older adults Psychol Aging 2007 22 1 52 55 17385982 10.1037/0882-7974.22.1.52

[B7] Smits CH de Lange J Droes RM Meiland F Vernooij-Dassen M Pot AM Effects of combined intervention programmes for people with dementia living at home and their caregivers: a systematic review Int J Geriatr Psychiatry 2007 22 12 1181 1193 10.1002/gps.1805 17457793

[B8] Romero B Wenz M Concept and effectiveness of a treatment program for patients with dementia and their relatives. Results from the bad aibling Alzheimer disease therapy center Z Gerontol Geriatr 2002 35 2 118 128 10.1007/s003910200016 12080575

[B9] Teri L Logsdon RG Uomoto J McCurry SM Behavioral treatment of depression in dementia patients: a controlled clinical trial J Gerontol B Psychol Sci Soc Sci 1997 52 4 P159 166 9224439 10.1093/geronb/52b.4.p159

[B10] Teri L Gibbons LE McCurry SM Logsdon RG Buchner DM Barlow WE Kukull WA LaCroix AZ McCormick W Larson EB Exercise plus behavioral management in patients with Alzheimer disease: a randomized controlled trial JAMA 2003 290 15 2015 2022 10.1001/jama.290.15.2015 14559955

[B11] Heyn P Abreu BC Ottenbacher KJ The effects of exercise training on elderly persons with cognitive impairment and dementia: a meta-analysis Arch Phys Med Rehabil 2004 85 10 1694 1704 10.1016/j.apmr.2004.03.019 15468033

[B12] Eggermont L Swaab D Luiten P Scherder E Exercise, cognition and Alzheimer's disease: more is not necessarily better Neurosci Biobehav Rev 2006 30 4 562 575 10.1016/j.neubiorev.2005.10.004 16359729

[B13] Rolland Y Pillard F Klapouszczak A Reynish E Thomas D Andrieu S Riviere D Vellas B Exercise program for nursing home residents with Alzheimer's disease: a 1-year randomized, controlled trial J Am Geriatr Soc 2007 55 2 158 165 10.1111/j.1532-5415.2007.01035.x 17302650

[B14] Potter R Ellard D Rees K Thorogood M A systematic review of the effects of physical activity on physical functioning, quality of life and depression in older people with dementia Int J Geriatr Psychiatry 2011 26 10 1000 1011 10.1002/gps.2641 21905096

[B15] Quinn C Clare L Woods B The impact of the quality of relationship on the experiences and wellbeing of caregivers of people with dementia: a systematic review Aging Ment Health 2009 13 2 143 154 10.1080/13607860802459799 19347681

[B16] Vliegendhart L Van der Mark C Thuis bewegen, houdt depressie tegen Handleiding 2009 Kenniskring Transities in Zorg: Rotterdam University

[B17] Ware J Snow K Kosinski M Gandek B Health survey: manual and interpretation guide 1993 Boston Mass: Health Institute NEMC

[B18] Van der Zee K Sanderma R Het meten van de algemene gezondheidstoestand met de RAND-36. Een handleiding 1993 Groningen: Noordelijk Centrum voor Gezondheidsvraagstukken

[B19] Bergner M Bobbitt RA Pollard WE Martin DP Gilson BS The sickness impact profile: validation of a health status measure Med Care 1976 14 1 57 67 10.1097/00005650-197601000-00006 950811

[B20] Luttik A Jacobs H Witte LPd Een Nederlandse versie van de sickness impact profile (Dutch version of the sickness impact profile) 1985 Vakgroep Huisartsgeneeskunde: Rijksuniversiteit Utrecht

[B21] Alexopoulos GS Abrams RC Young RC Shamoian CA Cornell scale for depression in dementia Biol Psychiatry 1988 23 3 271 284 10.1016/0006-3223(88)90038-8 3337862

[B22] Dröes R Cornell scale for depression in dementia. Nederlandse vertaling 1993 Amsterdam: Vrije Universiteit, Vakgroep Psychiatrie

[B23] Frijters D Achterberg W Hirdes JP Fries BE Morris JN Steel K Integrated health information system based on resident assessment instruments Tijdschr Gerontol Geriatr 2001 32 1 8 16 11293844

[B24] Morris J Fries B Bernabei R Steel K Ikegami N Carpenter G Gilgen R DuPasquier J Frijters D Henrard J RAI-Home Care (RAI-HC) assessment manual for version 2.0 2000 Marblehead, MA: Opus Communications

[B25] Morris JN Fries BE Steel K Ikegami N Bernabei R Carpenter GI Gilgen R Hirdes JP Topinkova E Comprehensive clinical assessment in community setting: applicability of the MDS-HC J Am Geriatr Soc 1997 45 8 1017 1024 9256857 10.1111/j.1532-5415.1997.tb02975.x

[B26] Sheikh J Yesavage J Geriatric depression scale (GDS): recent evidence and development of a shorter version Clin Gerontologist: J Aging Mental Health 1986 5 1-2 165 173

[B27] Kok R Heeren T Van Hemert A De geriatric depression scale Tijdschrift voor Psychiatrie 1993 35 6 416 421

[B28] Teri L Truax P Logsdon R Uomoto J Zarit S Vitaliano P Assessment of behavioral problems in dementia: the revised memory and behavior problems checklist Psychol Aging 1992 7 4 622 631 1466831 10.1037//0882-7974.7.4.622

[B29] Teunisse S de Haan R Walstra GJM de Rooij SEJA Zwart M Behavioural problems in mild dementia: clinical relevance and methodological evaluation of the revised memory and behavioural problems checklist PhD Thesis Clinimetrics in dementia 1997 Universiteit van Amsterdam

[B30] Van Someren EJ Actigraphic monitoring of movement and rest-activity rhythms in aging, Alzheimer's disease, and Parkinson's disease IEEE Trans Rehabil Eng 1997 5 4 394 398 10.1109/86.650297 9422465

[B31] Reisberg B Ferris SH de Leon MJ Crook T The global deterioration scale for assessment of primary degenerative dementia Am J Psychiatry 1982 139 9 1136 1139 7114305 10.1176/ajp.139.9.1136

[B32] Folstein MF Folstein SE McHugh PR "Mini-mental state". A practical method for grading the cognitive state of patients for the clinician J Psychiatr Res 1975 12 3 189 198 10.1016/0022-3956(75)90026-6 1202204

[B33] Kok R Verhey F Dutch translation of the mini mental state examination (Folstein et al., 1975) 2002

[B34] Lindeboom B Jonker C De Amsterdamse dementia screeningtest: handleiding 1989 Lisse: Swets and Zeitlinger

[B35] Wilson BA Alderman N Burgess P Emslie H Evans J The behavioural assessment of the dysexecutive syndrome 1996 Thames Valley Test Company: Flempton BSE

[B36] War Department AGsO Army individual test battery: manual of directions and scoring 1944 Washington, DC

[B37] Wechsler D Wechsler memory scale revised 1987 The Psychological Corporation HBJ, Inc

[B38] Wilson B Cockburn J Baddeley A The rivermead behavioural memory test 1985 Company, RTVT

[B39] Van Balen H Wimmers M Rivermead behavioural memory test. Normeringsgegevens voor Nederland en Vlaanderen 1993 Lisse: Swets Test Services

[B40] Bizzarro A Marra C Acciarri A Valenza A Tiziano FD Brahe C Masullo C Apolipoprotein E epsilon4 allele differentiates the clinical response to donepezil in Alzheimer's disease Dement Geriatr Cogn Disord 2005 20 4 254 261 10.1159/000087371 16103669

[B41] Radloff L The CES-D Scale: a self-report depression scale for research in the general population Appl Psych Meas 1977 1 385 401 10.1177/014662167700100306

[B42] Bouma RA J Sanderman R Van Sonderen FLP Het meten van symptomen van depressie met de CES-D. Een handleiding 1995 Groningen: Noordelijk Centrum voor Gezondheidsvraagstukken, Rijksuniversiteit Groningen

[B43] Pot AM Deeg DJ van Dyck R Jonker C Psychological distress of caregivers: the mediator effect of caregiving appraisal Patient Educ Couns 1998 34 1 43 51 10.1016/S0738-3991(98)00048-2 9697556

[B44] Pot AM van Dyck R Deeg DJ Perceived stress caused by informal caregiving. Construction of a scale Tijdschr Gerontol Geriatr 1995 26 5 214 219 8750982

[B45] Kirschbaum C Hellhammer DH Salivary cortisol in psychoneuroendocrine research: recent developments and applications Psychoneuroendocrinology 1994 19 4 313 333 10.1016/0306-4530(94)90013-2 8047637

[B46] Pruessner JC Wolf OT Hellhammer DH Buske-Kirschbaum A von Auer K Jobst S Kaspers F Kirschbaum C Free cortisol levels after awakening: a reliable biological marker for the assessment of adrenocortical activity Life Sci 1997 61 26 2539 2549 10.1016/S0024-3205(97)01008-4 9416776

[B47] Liang K Zeger S Longitudinal data analysis using generalized lineair models Biometrika 1986 73 1 13 2 10.1093/biomet/73.1.13

